# Integrative functional and molecular characterization of Bali bull semen and its relationship with reproductive performance

**DOI:** 10.14202/vetworld.2026.1447-1458

**Published:** 2026-04-12

**Authors:** Hendri Hendri, Ananda Ananda, Erni Damayanti, Herry Sonjaya, Zulfi Nur Amrina Rosyada, Mirni Lamid, Moh Anam Al Arif, Widya Paramita Lokapirnasari, Ashariah Hapila, Tulus Maulana, Hikmayani Iskandar

**Affiliations:** 1 Department of Animal Production Technology, Faculty of Animal Science, Universitas Andalas, Padang, Indonesia; 2 Department of Animal Production, Faculty of Animal Science, Universitas Hasanuddin, Makassar, Indonesia; 3 Division of Animal Husbandry, Faculty of Veterinary Medicine, Universitas Airlangga, Surabaya, Indonesia; 4 Department of Animal Nutrition and Feed Science, Faculty of Animal Science, Universitas Hasanuddin, Makassar, Indonesia; 5 Research Center for Applied Zoology, National Research and Innovation Agency (BRIN), Bogor, Indonesia

**Keywords:** acrosome integrity, Bali cattle, canonical correlation analysis, chromatin integrity, computer-assisted sperm analysis, DNA integrity, protamine integrity, semen quality

## Abstract

**Background and Aim:**

Bali cattle (*Bos javanicus*) are an important indigenous genetic resource in Indonesia, characterized by high adaptability and reproductive efficiency. However, conventional semen evaluation based on motility and morphology often fails to accurately predict bull fertility. Integrating functional sperm analysis with molecular quality indicators may provide a more reliable assessment of reproductive potential. This study aimed to perform an integrative evaluation of Bali bull semen by combining computer-assisted sperm analysis (CASA) parameters with molecular sperm quality indicators, including acrosome integrity, DNA integrity, and protamine status, and to determine their multivariate relationships using canonical correlation analysis (CCA).

**Materials and Methods:**

Frozen–thawed semen samples were collected from ten Bali bulls maintained at a Regional Artificial Insemination Center. Functional sperm parameters were evaluated using CASA, including total motility, progressive motility, velocity parameters (velocity average path, velocity curved line, and velocity straight line), linearity, straightness, wobble, amplitude of lateral head displacement, and beat cross frequency. Acrosome integrity was assessed using fluorescein isothiocyanate-conjugated peanut agglutinin staining, DNA integrity was evaluated using acridine orange staining, and protamine integrity was determined using chromomycin A3 staining. Data were analyzed using one-way analysis of variance followed by Tukey’s test, and multivariate relationships between functional and molecular parameters were examined using CCA.

**Results:**

CASA evaluation demonstrated inter-individual variation in semen quality, with total motility ranging from 49.80% to 60.48%. Acrosome integrity remained high across all bulls (85.03–95.59%), while DNA integrity (96.41%–98.55%) and protamine status (90.35%–97.04%) indicated well-preserved chromatin structure. The first canonical function showed a strong correlation between functional and molecular variables (r = 0.906), although the association was not statistically significant (p = 0.134). The canonical plot revealed a consistent positive trend, suggesting coordinated variation between sperm kinematics and chromatin integrity markers.

**Conclusion:**

The integrative evaluation of CASA-derived kinematic parameters with molecular sperm quality indicators provides a more comprehensive assessment of Bali bull fertility than conventional semen analysis alone. The strong but non-significant canonical correlation suggests a biologically plausible relationship between sperm motility and chromatin stability. This study provides preliminary evidence supporting the use of combined functional and molecular semen evaluation to improve bull selection, enhance artificial insemination efficiency, and support genetic conservation of Bali cattle. Further studies with larger sample sizes and fertility-validated outcomes are required to confirm these findings.

## INTRODUCTION

Bali cattle (*Bos javanicus*) are one of the most important indigenous cattle breeds in Indonesia, valued for their adaptability, fertility, and resilience in marginal environments [1, 2]. Representing approximately 34.9% of Indonesia’s local cattle population, with an estimated population of around 11 million head, Bali cattle contribute significantly to smallholder farming systems and national meat production [2, 3]. Their reproductive efficiency is a key trait supporting their sustainability, with studies reporting normal reproductive function in more than 70% of cows and pregnancy rates reaching up to 80% under farm conditions [4, 5]. Despite these advantages, reproductive performance is often challenged by infertility, repeat breeding, and reproductive tract infections, which can impair conception and calving rates [6, 7]. Therefore, a comprehensive evaluation of male fertility, particularly bull semen quality, is essential for improving reproductive efficiency and ensuring the genetic conservation of Bali cattle. Traditionally, semen quality assessments have relied on macroscopic and microscopic traits such as volume, motility, concentration, and morphology. However, these parameters are often insufficient to predict fertility accurately, as bulls with apparently normal semen profiles may still exhibit subfertility [8, 9]. Advances in computer-assisted sperm analysis (CASA) have provided a more objective and quantitative framework for evaluating sperm kinematics, including velocity average path (VAP), velocity straight line (VSL), velocity curvilinear (VCL), linearity (LIN), and straightness (STR) [10, 11]. CASA reduces subjectivity and enhances reproducibility compared to conventional microscopic evaluation [12, 13]. Moreover, CASA-based motility parameters have been strongly correlated with fertilization rates in cattle, making them valuable predictors of reproductive performance [14, 15]. Given the limited molecular-level data on Bali bull sperm chromatin, particularly protamine packaging and DNA stability, compared with taurine breeds, this study fills a critical gap for genetic conservation, where high chromatin integrity is essential to minimize the transmission of subfertile traits in smallholder systems.

Beyond functional assessment, molecular markers are increasingly recognized as critical determinants of sperm quality and fertility. Protamines, the nuclear proteins that replace histones during spermatogenesis, are essential for sperm chromatin compaction and DNA protection. Protamine deficiency results in poor chromatin packaging, higher DNA fragmentation, and reduced fertility outcomes [16–18]. Chromomycin A3 (CMA3) staining has been widely used to evaluate protamine content, with higher staining intensities indicating inadequate protamination and reduced fertility potential [19, 20]. Similarly, DNA integrity is a crucial parameter of male fertility, as elevated levels are associated with impaired fertilization, compromised embryo quality, and increased miscarriage rates [21, 22]. Acridine orange staining has been applied extensively to assess DNA integrity, distinguishing between intact double-stranded and damaged single-stranded DNA [23, 24]. In addition, acrosome integrity is indispensable for fertilization, as defects in this structure impair the sperm’s ability to penetrate the oocyte [25, 26]. Together, these molecular parameters provide insights into chromatin stability and fertilizing capacity that extend beyond conventional semen evaluation. Integrating functional and molecular assessments represents a powerful approach to characterizing semen quality more comprehensively. Previous studies have reported correlations between CASA-derived sperm kinematic parameters and molecular indices, including DNA integrity and protamine content, indicating that sperm with higher motility generally possess superior DNA integrity and chromatin stability [27, 28]. Furthermore, oxidative stress, which contributes to DNA damage, has been linked to reduced sperm motility, emphasizing the interconnectedness of functional and molecular attributes in determining fertility [29]. Advanced statistical methods such as canonical correlation analysis (CCA) provide an opportunity to evaluate the complex relationships between these functional and molecular variables simultaneously, offering a more integrative understanding of male fertility potential [30].

The evaluation of bull fertility in artificial insemination programs is still largely dependent on conventional semen quality traits and field fertility outcomes, although these measures do not always provide sufficiently accurate prediction of reproductive performance. Previous studies have shown that the quality of frozen–thawed semen is related to fertility rate after distribution in breeding programs, confirming that semen quality assessment remains central to sire selection [31]. In addition, sire fertility has been shown to influence the reproductive performance of offspring and overall herd productivity, highlighting the broader genetic and production consequences of selecting bulls with suboptimal fertility [32]. However, these approaches mainly emphasize conventional semen evaluation and fertility-associated outcomes, without adequately integrating functional sperm kinematics with molecular indicators of sperm quality. In Bali cattle, this gap is even more evident because studies examining semen quality have generally focused on isolated functional or reproductive outcomes, while a combined assessment of sperm motion characteristics, acrosome integrity, protamine status, and DNA integrity remains limited. Therefore, a more comprehensive and integrative semen evaluation framework is needed to improve fertility prediction, strengthen bull selection, and support breeding efficiency and genetic conservation in Bali cattle.

Therefore, the present study was undertaken to provide an integrative characterization of Bali bull semen by combining functional sperm evaluation with molecular sperm quality assessment. Functional semen characteristics were assessed using CASA, including motility and sperm kinematic parameters, while molecular sperm quality was evaluated through acrosome integrity, protamine status, and DNA integrity. Furthermore, CCA was applied to examine the multivariate relationships between functional semen traits and molecular sperm quality indices in order to obtain a more comprehensive understanding of semen quality. Through this combined approach, the study aimed to determine whether sperm movement characteristics are associated with chromatin stability and structural integrity, and to explore the biological relevance of these interactions in Bali bull fertility assessment. Ultimately, the findings were expected to contribute to the development of more accurate selection criteria for bulls used in artificial insemination programs and to support the sustainable improvement and genetic conservation of Bali cattle.

## MATERIALS AND METHODS

### Ethical approval

All procedures involving animals were reviewed and approved by the Animal Ethics Committee of Universitas Airlangga, Indonesia (Approval No. 1.KEH.107.07.2025, dated 10 July 2025). The study was conducted in accordance with the national guidelines for the care and use of animals in research and followed the institutional standard operating procedures for handling breeding bulls. Semen samples used in this study were obtained from routine semen collection performed at the Regional Artificial Insemination Center, South Sulawesi, as part of the regular artificial insemination program, and no additional invasive procedures were performed specifically for research purposes. All bulls were maintained under standard management conditions, including proper housing, feeding, and veterinary supervision, to ensure animal welfare throughout the study period. Handling of animals during semen collection was carried out by trained personnel using an artificial vagina according to the established protocol of the artificial insemination center, minimizing stress and discomfort to the animals. Transportation, storage, and laboratory analysis of frozen semen were conducted following biosafety and laboratory animal-use regulations. The study complied with Indonesian regulations on animal welfare in research and with internationally accepted principles for the ethical use of animals in scientific studies.

### Study period and location

This study was conducted from March to August 2025. Semen from Bali bulls was collected twice weekly in the morning using an artificial vagina at the Regional Artificial Insemination Center (RAIC), South Sulawesi, Indonesia. Frozen semen samples were stored in liquid nitrogen containers at −196°C and transported under cryogenic conditions to the Veterinary and Zoology Laboratory, National Research and Innovation Agency (BRIN), for further analysis.

### Experimental design and animal care

Frozen semen samples were obtained from ten Bali bulls aged 8–12 years. Semen quality was initially evaluated based on volume, concentration, motility, and morphology. Only ejaculates meeting the minimum quality standards (≥70% motility and ≥80% normal morphology) were included in the study. Bulls were maintained according to the standard operating procedures of the South Sulawesi RAIC. Each bull was housed individually in a 2.5 × 2 m pen equipped with feeding and drinking containers. Animals were fed fresh forage equivalent to 10% of body weight and 2 kg of concentrate twice daily, in the morning and evening, and water was provided *ad libitum*.

### Frozen–thawed semen

Frozen semen straws produced in 2025 were used in this study. The straws were thawed in a water bath at 37°C for 30 s. After drying, both ends of the straw were cut and the semen was transferred into a pre-warmed 1.5 mL Eppendorf tube. The thawed semen was incubated at 37°C for 5–10 min before further evaluation.

## CASA

Sperm motility and kinematic parameters were evaluated using CASA (SpermaVision™ 3.7.8, Minitube, Germany) connected to a phase-contrast microscope (Axio Scope A1, Carl Zeiss, Germany) equipped with a heated stage set at 38°C. A 5 µL aliquot of thawed semen was placed on a pre-warmed slide and covered with an 18 × 18 mm coverslip. Eight randomly selected fields were analyzed per sample, with at least 1000 spermatozoa evaluated. Recorded parameters included total motility, progressive motility, distance average path (DAP), distance curve linear (DCL), distance straight line (DSL), velocity average path, velocity curve linear (VCL), velocity straight line (VSL), straightness (STR), linearity (LIN), wobble (WOB), average lateral head displacement (ALH), and beat cross frequency (BCF). Motility observations were performed at 200× magnification, and mean values were calculated from approximately 1000 spermatozoa per sample [33].

### Assessment of acrosome integrity

**Figure 1:**
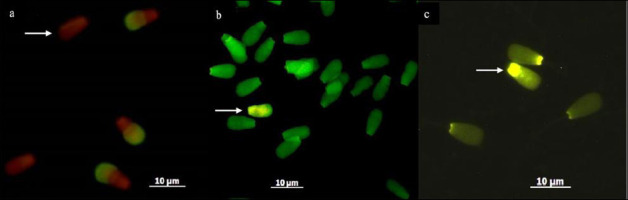
Sperm acrosome integrity, DNA integrity, and protamine integrity assessment under fluorescence microscopy (60× magnification, Imager Z2, Carl Zeiss, Germany). (a) Acrosome integrity assessed using FITC-PNA staining; arrow indicates sperm with loss of acrosome (no FITC-PNA staining). (b) DNA integrity assessed using acridine orange staining; arrow indicates sperm with green to yellow fluorescence in the head (intact DNA). (c) Protamine integrity assessed using chromomycin A3; arrow indicates dull green round-headed sperm cells showing protamine deficiency.

Acrosome integrity was evaluated using fluorescein isothiocyanate-conjugated peanut agglutinin staining (FITC-PNA; Sigma-Aldrich, USA). Semen smears were fixed in 96% ethanol for 10 min at 25°C and air-dried. Slides were incubated with 30 µL FITC-PNA (100 µg/mL) for 30 min at 37°C in the dark, followed by counterstaining with 5 µL propidium iodide (1 µg/mL) for 5 min. Slides were rinsed with phosphate-buffered saline, mounted, and examined under a fluorescence microscope (AxioPhot Zeiss, Oberkochen, Germany; 490/530 nm excitation filter) using FITC and PI filter sets. A minimum of 200 spermatozoa per sample was evaluated. Spermatozoa showing green fluorescence in the acrosomal region were considered intact, whereas those showing red fluorescence or no green signal were classified as acrosome-damaged (Figure 1a) [34].

### Evaluation of sperm DNA integrity

DNA integrity was assessed using acridine orange staining following the method described by Said *et al*. [35]. A semen smear was prepared on a clean glass slide and air-dried over a gentle flame. The smear was fixed in Carnoy’s solution for 2 h, rinsed with distilled water, and air-dried. The fixed sample was stained with acridine orange for 5 min, rinsed, mounted with a coverslip, sealed, and examined under a fluorescence microscope to evaluate DNA integrity (Figure 1b).

### Assessment of protamine status

Protamine status was evaluated using the CMA3 staining technique [34]. Sperm samples were washed twice in phosphate-buffered saline by centrifugation, fixed in Carnoy’s solution, and spread on APES-treated slides. Slides were stained with CMA3 solution (0.25 mg/mL) in McIlvain buffer for 30 min, rinsed, air-dried, and mounted with antifade solution (Fluoprep, BioMérieux, France). Observations were performed using a fluorescence microscope at 460–470 nm. Spermatozoa with protamine deficiency appeared bright yellow, whereas spermatozoa with normal protamine content appeared dull yellow (Figure 1c).

### Statistical analysis

Data are expressed as mean ± standard deviation. Differences among bulls were analyzed using one-way analysis of variance followed by Tukey’s post hoc test. Relationships between kinematic parameters and molecular sperm quality indices were evaluated using CCA in R Studio (version 4.3.2). Statistical significance was set at p < 0.05.

## RESULTS

### Semen quality based on CASA parameters

The evaluation revealed clear inter-bull variation in semen kinematic quality. Among all individuals, Arjuna and Celebes showed superior sperm performance, as evidenced by higher progressive motility and increased velocity parameters (VAP, VCL, and VSL) (Table 1). These findings indicate a more efficient and stable sperm trajectory, reflecting optimal flagellar activity and energy utilization. Such characteristics suggest greater fertilizing ability, making these bulls suitable candidates for use in AI programs. In contrast, Rowa, Bima, Rewa, Sinyo, and Singo exhibited comparatively lower semen quality, particularly with respect to progressive motility and sperm velocity, which may reduce their fertilization potential. Kajuara and Lewa showed moderate total motility but lower progressivity and movement efficiency compared with Arjuna and Celebes. Overall, these results highlight the importance of sperm kinematic parameters as objective indicators of male fertility potential. The use of CASA-based sperm motion analysis provides a reliable approach for identifying superior sires and improving reproductive efficiency in breeding programs.

**Table 1:** Semen quality based on computer-assisted sperm analysis parameters of Bali bull (n = 10).

Parameter	Celebes	Rowa	Bima	Arjuna	Kajuara	Lewa	Maiwa	Rewa	Sinyo	Singo
% Motile	55.93 ± 4.94	53.26 ± 2.11	50.46 ± 1.20	49.80 ± 7.28	54.67 ± 1.18	60.48 ± 2.75	53.05 ± 1.95	53.80 ± 3.58	54.54 ± 1.45	53.98 ± 3.88
% Progressive	53.71 ± 2.49^c^	42.43 ± 4.81^a^	43.98 ± 6.08^a^	51.07 ± 2.35^bc^	46.91 ± 4.55^abc^	47.45 ± 3.77^abc^	47.36 ± 2.67^abc^	44.42 ± 3.16^ab^	43.30 ± 0.99^a^	45.79 ± 2.65^ab^
DAP	38.81 ± 1.36^d^	28.75 ± 3.26^ab^	28.11 ± 4.50^ab^	36.38 ± 1.99^cd^	26.27 ± 2.55^a^	27.81 ± 1.88^ab^	32.96 ± 3.01^bc^	29.80 ± 2.03^ab^	29.30 ± 1.20^ab^	27.48 ± 4.41^a^
DCL	68.07 ± 3.24	58.38 ± 29.35	57.64 ± 29.99	59.13 ± 11.44	37.93 ± 3.19	39.54 ± 3.34	43.32 ± 1.53	41.99 ± 3.42	41.17 ± 3.41	38.14 ± 5.88
DSL	26.44 ± 3.06^cd^	20.24 ± 2.26^ab^	18.92 ± 2.31^ab^	31.39 ± 6.63^d^	19.11 ± 1.85^ab^	18.66 ± 1.06^ab^	23.81 ± 1.48^bc^	21.27 ± 2.34^abc^	21.10 ± 1.54^abc^	17.71 ± 2.96^a^
VAP	86.55 ± 8.56^bc^	69.77 ± 4.88^ab^	69.47 ± 9.86^ab^	91.45 ± 15.92^c^	62.05 ± 7.94^a^	64.82 ± 4.58^a^	74.34 ± 4.51^abc^	74.11 ± 6.16^abc^	72.04 ± 6.40^ab^	60.46 ± 17.54^a^
VCL	119.80 ± 5.48	101.93 ± 11.51	105.94 ± 18.79	124.16 ± 6.88	95.06 ± 7.51	95.10 ± 6.56	108.34 ± 9.61	107.98 ± 10.96	98.91 ± 10.55	97.14 ± 25.05
VSL	62.84 ± 6.31^bc^	49.11 ± 2.52^a^	44.74 ± 4.84^a^	72.10 ± 15.51^c^	45.40 ± 4.22^a^	45.32 ± 3.96^a^	54.16 ± 1.61^ab^	52.12 ± 5.68^ab^	45.85 ± 2.37^a^	47.90 ± 8.44^a^
STR	77.00 ± 6.08^cd^	73.00 ± 1.73^bcd^	64.33 ± 4.04^a^	79.66 ± 7.02^d^	67.33 ± 6.65^ab^	65.66 ± 0.57^ab^	76.00 ± 2.64^cd^	70.33 ± 1.52^abc^	69.00 ± 6.08^abc^	66.33 ± 1.52^ab^
LIN	56.66 ± 0.57^d^	50.00 ± 2.00^abc^	55.33 ± 7.76^cd^	54.33 ± 2.08^d^	52.66 ± 3.05^bcd^	46.00 ± 1.00^a^	55.33 ± 3.21^cd^	50.66 ± 2.88^abcd^	54.00 ± 1.73^bcd^	48.00 ± 1.00^ab^
WOB	72.33 ± 3.78	73.66 ± 4.16	73.33 ± 4.04	69.33 ± 3.21	74.66 ± 4.93	74.66 ± 1.15	76.33 ± 2.08	73.66 ± 1.52	72.33 ± 4.04	76.00 ± 2.00
ALH	5.09 ± 0.24^bc^	5.71 ± 0.17^cd^	6.07 ± 0.71^d^	5.48 ± 0.16^bc^	4.94 ± 0.37^bc^	5.57 ± 0.28^bc^	4.78 ± 0.50^b^	5.76 ± 0.27^cd^	5.20 ± 0.68^bc^	3.88 ± 0.41^a^
BCF	31.62 ± 2.58^c^	23.03 ± 3.02^ab^	22.78 ± 3.05^ab^	30.96 ± 2.55^c^	23.33 ± 4.34^ab^	21.79 ± 0.48^a^	27.44 ± 1.93^bc^	24.29 ± 3.31^ab^	22.32 ± 2.01^a^	21.75 ± 0.91^a^

DAP = distance average path (µm); DCL = distance curved line (µm); DSL = distance straight line (µm); VAP = velocity average path (µm/s); VCL = velocity curved line (µm/s); VSL = velocity straight line (µm/s); STR = straightness (%); LIN = linearity (%); WOB = wobble (%); ALH = amplitude of lateral head displacement (µm); BCF = beat cross frequency (Hz).

Different letters within the same row indicate significant difference (p < 0.05).

### Sperm acrosomal integrity, DNA integrity, and protamine integrity

Acrosomal integrity remained high in all bulls, ranging from 85.03% to 95.59%, with the highest values observed in Kajuara and Lewa (>95%), indicating better membrane stability and potentially higher fertilization capacity (Table 2). DNA integrity values were consistently high (96.41%–98.55%), suggesting a very low level of DNA damage among the samples. Protamine status, which reflects the degree of chromatin condensation, also showed high values (90.35–97.04%). The highest values were recorded in Arjuna (97.04%) and Bima (96.61%), indicating well-condensed chromatin and improved protection of spermatozoa from genetic damage.

**Table 2:** Sperm acrosomal integrity, DNA integrity, and protamine integrity.

Parameter	Celebes	Rowa	Bima	Arjuna	Kajuara	Lewa	Maiwa	Rewa	Sinyo	Singo
Acrosomal integrity	88.21 ± 5.38^ab^	85.03 ± 1.65^a^	89.84 ± 6.25^abc^	88.00 ± 1.57^ab^	95.13 ± 3.05^c^	95.59 ± 1.20^c^	91.20 ± 0.98^abc^	90.67 ± 2.14^abc^	92.46 ± 3.35^bc^	84.76 ± 4.00^a^
DNA integrity	98.50 ± 1.51	98.24 ± 1.46	98.48 ± 0.79	98.41 ± 0.63	98.55 ± 1.74	96.71 ± 0.40	97.24 ± 1.26	97.38 ± 1.40	96.41 ± 1.22	97.78 ± 1.82
Protamine integrity	95.17 ± 3.62	90.35 ± 3.58	96.61 ± 2.67	97.04 ± 1.22	95.71 ± 1.13	93.85 ± 2.20	93.75 ± 0.61	96.68 ± 0.62	95.10 ± 0.86	94.32 ± 4.29

### Correlation between sperm quality and acrosome integrity, DNA integrity, and protamine integrity

**Figure 2:**
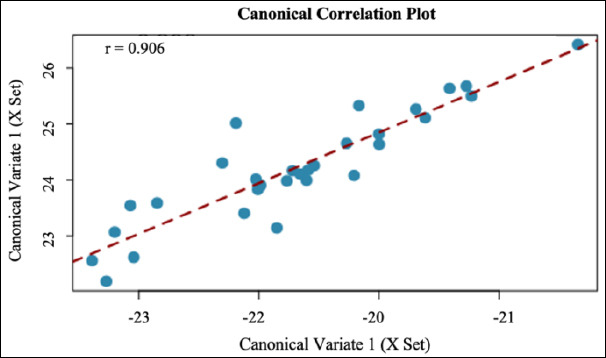
Correlation between sperm quality and sperm acrosome integrity, DNA integrity, and protamine integrity.

The canonical correlation plot demonstrated the relationship between canonical variates derived from CASA-based sperm kinematic parameters and molecular sperm quality indicators. The analysis produced a high canonical correlation coefficient for the first canonical function (r = 0.906), indicating a strong linear association between the two variable sets (Figure 2). However, this relationship was not statistically significant (*p* = 0.134), most likely due to the limited statistical power resulting from the small sample size (n = 10). The distribution of the data points showed a consistent positive trend, suggesting coordinated variation between sperm motility characteristics and molecular integrity parameters.

## DISCUSSION

### Semen quality based on CASA parameters

The CASA evaluation of Bali bull semen shows that total motility ranged from 49.80% to 60.48%, with mean values exceeding the minimum threshold generally required for artificial insemination. These results indicate that the semen quality of Bali bulls was adequate to support fertilization. The observed variation in kinematic parameters, including VAP, VSL, and VCL, reflects individual differences in sperm movement patterns. In addition, variations in linearity (LIN) and straightness (STR) suggest differences in the efficiency and directionality of sperm trajectory among individuals.

CASA has been widely recognized as a reliable and objective technique for assessing sperm motility compared with conventional microscopic evaluation, which is often affected by subjectivity and observer variation [14]. Sperm motility is a critical determinant of male fertility, as spermatozoa must exhibit adequate progressive movement to successfully traverse the female reproductive tract and reach the oocyte for fertilization [36]. Previous studies have reported that CASA-derived motility parameters, such as VCL, VSL, and VAP, are significantly correlated with fertilization success in cattle [15, 37]. The present findings demonstrate that Bali bulls possess motility values within the fertile range, supporting their suitability for use as sires in breeding programs.

The variability in CASA parameters observed among individuals in this study indicates the influence of both genetic and non-genetic factors. Environmental conditions, management practices, and physiological status are known to contribute to differences in semen quality [38]. Similar findings have been reported in Bonsmara and Nguni bulls, where CASA analysis revealed significant breed-related variation in sperm morphometry and motility [39]. These observations suggest that the moderate variability detected in Bali bulls is consistent with patterns reported in both indigenous and commercial cattle breeds.

Bull age is another factor that may affect semen motility. Previous reports have shown that total and progressive motility tend to decline in bulls older than 7–8 years and may be accompanied by increased DNA damage, which can reduce fertility [34, 40]. Although age was not specifically analyzed in the present study, the range of CASA values obtained may partly reflect differences in physiological maturity among the bulls. In addition, environmental stress, seasonal changes, and semen handling procedures may also influence motility parameters [41].

Overall, the CASA-based evaluation demonstrated that the motility and kinematic parameters of Bali bull semen met the requirements for artificial insemination. These findings confirm the usefulness of CASA as a tool for predicting reproductive performance and support its application for more accurate assessment of bull fertility. Incorporating CASA into breeding soundness evaluation may improve the identification of subfertile bulls and enhance the efficiency of artificial insemination programs in Bali cattle.

### Molecular indices of sperm quality

The molecular evaluation of Bali bull semen showed consistently high values for all assessed parameters. The proportion of spermatozoa with intact acrosomes exceeded 90%, indicating that most sperm retained the structural integrity required for successful oocyte penetration. This observation is consistent with previous reports stating that acrosome integrity is essential for the acrosome reaction, which plays a critical role during fertilization [25]. In addition, high acrosomal integrity has been associated with proper chromatin maturation and improved fertilization ability [26]. The relatively high post-thaw acrosome integrity observed in the present study may be explained by several factors, including the inherent resistance of Bali cattle sperm to cryopreservation stress, the use of selected AI bulls with proven reproductive performance, and the sensitivity of the fluorescence staining method, which may preferentially detect structurally intact acrosomes. Furthermore, the use of frozen semen that met acceptable post-thaw quality standards may have contributed to better preservation of acrosomal membranes. Taken together, these findings indicate that Bali bull sperm possess strong structural stability suitable for use in artificial insemination and other assisted reproductive techniques, although confirmation using additional functional and fertility-based tests is still required.

Protamine status, evaluated using CMA3 staining, also showed favorable results, with more than 95% of spermatozoa exhibiting proper chromatin packaging, while only a small proportion showed abnormal condensation during spermiogenesis, which is essential for protecting DNA from damage and oxidative stress [42, 43]. Previous studies have consistently reported that sperm with protamine deficiency are more susceptible to DNA damage and are associated with reduced fertility [18, 44]. Therefore, the low CMA3 positivity observed in Bali bulls indicates high chromatin stability and supports their potential fertility and suitability for breeding programs. In addition, CMA3-based evaluation has been widely used as a predictor of fertilization success in both animal and human reproductive studies [45], further supporting the relevance of this molecular marker.

Similarly, the acridine orange test revealed very high levels of DNA integrity in Bali bull sperm, with more than 96% of spermatozoa showing intact double-stranded DNA. This finding is consistent with previous studies reporting that lower DNA damage is associated with higher fertilization rates, improved embryo development, and higher pregnancy success in assisted reproductive technologies [21, 22]. Conversely, increased DNA damage has been linked to poor embryo quality and a higher risk of pregnancy loss [46]. Therefore, the high DNA integrity observed in the present study indicates strong genetic stability of Bali bull sperm, which may positively influence reproductive outcomes.

Overall, the molecular indices evaluated in this study, including acrosome integrity, protamine status, and DNA integrity, showed a consistent pattern of high sperm quality in Bali bulls. The combined evaluation of these parameters reflects strong chromatin stability in this breed and supports its importance as a valuable genetic resource for breeding and conservation programs. These findings also emphasize that molecular markers should be considered together with functional semen evaluation to obtain a more accurate assessment of fertility potential in bulls.

The relatively high acrosomal integrity, protamine status, and DNA integrity observed in the present study were higher than those reported in several previous studies on frozen–thawed semen of Bali bulls and other indigenous cattle breeds, in which post-thaw motility and membrane integrity were often reduced. These differences may be related to variation in bull selection, semen processing methods, or differences in evaluation techniques.

### CCA between functional and molecular parameters

The CCA in this study revealed a strong association between functional semen parameters measured by CASA and molecular indicators of sperm quality (Table 3). The first canonical function showed a high canonical correlation coefficient, indicating a strong linear relationship between composite sperm kinematic variables, particularly motility and velocity parameters (VAP, VSL, and VCL), and molecular traits such as acrosome integrity, protamine status, and DNA integrity. However, this relationship did not reach statistical significance, suggesting that the observed association should be interpreted cautiously and considered exploratory. The canonical plot shows a consistent directional trend between sperm motion characteristics and molecular integrity parameters, supporting a biologically meaningful interaction. Nevertheless, the limited sample size in the present study may have reduced the statistical power, and therefore these findings should be confirmed in larger populations, including fertility-validated data.

**Table 3:** Correlation between sperm quality and acrosomal integrity, DNA integrity, and protamine integrity.

Canonical Function	Canonical Correlation (r)	p-value
Function 1	0.906	0.134
Function 2	0.626	0.914
Function 3	0.479	0.918

The present study provides an integrative evaluation combining objective CASA-derived kinematic parameters with simultaneous assessment of acrosome integrity, protamine status determined by CMA3 staining, and DNA integrity assessed by acridine orange staining in frozen–thawed Bali bull semen, analyzed using a multivariate approach through CCA. Previous studies in Bali bulls have mainly focused on CASA-based sperm motility, seminal plasma composition, or individual molecular parameters, and these investigations have generally examined functional and molecular aspects separately [10, 37]. To the best of our knowledge, no previous study has simultaneously integrated sperm kinematic characteristics with chromatin-related quality parameters within a multivariate analytical framework to explore their interrelationships in Bali bulls.

The multivariate approach used in this study revealed a strong functional–molecular association, as indicated by the high canonical correlation in the first function, suggesting that sperm movement characteristics are closely related to chromatin stability and structural integrity. Compared with previous studies in indigenous cattle breeds, this integrative analysis may provide a more comprehensive model for predicting fertility, particularly in conservation-oriented breeding programs. Unlike conventional studies that evaluate either kinematic parameters or single molecular markers, the present approach links post-thaw functional performance with chromatin stability indicators that are critical for embryo development and maintenance of genetic quality in indigenous cattle populations.

These results support the concept that sperm motility should not be considered an isolated parameter, but rather as a trait closely related to chromatin organization and DNA stability. Earlier studies have shown that defective protamination and increased DNA damage may negatively affect sperm motility and fertilization capacity [27, 47]. In agreement with these findings, previous reports have demonstrated that CASA-derived velocity parameters, especially VAP and VCL, are associated with DNA quality, indicating that sperm with better motility generally possess more stable chromatin structure [48].

The integration of functional and molecular parameters using CCA provides a more comprehensive evaluation of semen quality than conventional methods. Molecular markers such as protamine status evaluated by CMA3 and DNA integrity assessed by acridine orange staining have been identified as sensitive indicators of fertility potential [18, 44], whereas CASA provides precise and objective measurements of sperm kinematics that are directly related to fertilization success [14, 15]. By combining these datasets, CCA allows the identification of hidden relationships between variables that may not be detected when each parameter is analyzed separately.

The canonical relationships observed in this study also have practical implications for breeding soundness evaluation in Bali bulls. Bulls that pass routine semen quality tests may still show poor fertility under field conditions, resulting in economic losses in artificial insemination programs [8]. The combined use of CASA and molecular sperm quality indices analyzed through CCA may improve the accuracy of fertility prediction and help to identify subfertile bulls before they are used in breeding programs.

From a biological perspective, the results suggest that proper chromatin packaging and intact DNA structure are necessary for maintaining optimal sperm motility. Oxidative stress and abnormal protamination can disrupt chromatin stability, leading to increased DNA damage and reduced sperm movement, as reported in both bovine and human studies [49]. The strong canonical loadings observed in the present study support this mechanism, indicating that deterioration of chromatin integrity may occur in parallel with reduced sperm motility.

Overall, these findings demonstrate the importance of applying multivariate statistical approaches such as CCA in the evaluation of bull fertility. The integration of functional kinematic parameters with molecular sperm quality indicators provides a more complete understanding of semen quality and may improve the selection of superior breeding bulls. This approach has potential to enhance the efficiency of artificial insemination programs and to support genetic conservation strategies in Bali cattle.

## CONCLUSION

The present study provided an integrative evaluation of Bali bull semen by combining functional sperm kinematic parameters obtained using CASA with molecular indicators of sperm quality, including acrosome integrity, protamine status, and DNA integrity. The results demonstrated that most bulls showed motility values within the acceptable range for artificial insemination, with clear inter-individual variation in kinematic parameters such as VAP, VSL, and VCL. Molecular assessment revealed consistently high acrosome integrity, high protamine stability, and low DNA damage, indicating good chromatin organization and structural integrity of spermatozoa. The CCA showed a strong but non-significant association between functional and molecular parameters, suggesting a biologically meaningful relationship between sperm motility and chromatin stability, although the limited sample size reduced statistical power.

From a practical perspective, these findings indicate that evaluation of semen quality based only on conventional parameters may be insufficient for accurate prediction of bull fertility. The combined use of CASA-derived kinematic analysis and molecular sperm quality markers provides a more comprehensive assessment of reproductive potential and may improve the selection of superior bulls for artificial insemination programs. Such an integrative approach is particularly important in Bali cattle, where maintaining reproductive efficiency is essential for both breeding productivity and genetic conservation.

A major strength of this study is the simultaneous assessment of functional and molecular sperm characteristics analyzed using a multivariate statistical approach, which allows a more holistic understanding of semen quality compared with conventional single-parameter evaluation. However, the study also has limitations, including the relatively small number of bulls and the absence of direct fertility data, which may explain the lack of statistical significance in the CCA. Therefore, the results should be interpreted cautiously and considered preliminary.

Future studies should include a larger population of bulls and incorporate field fertility outcomes to validate the relationships between kinematic parameters, chromatin stability, and reproductive performance. In addition, the inclusion of additional molecular markers related to oxidative stress, mitochondrial function, and sperm proteomics may further improve the accuracy of fertility prediction.

In conclusion, the integrative evaluation of functional sperm kinematics and molecular sperm quality provides a more reliable framework for assessing Bali bull fertility than conventional semen analysis alone. This approach has potential to improve bull selection, enhance the efficiency of artificial insemination programs, and support the long-term genetic conservation and sustainable utilization of Bali cattle.

## DATA AVAILABILITY

The data generated during the study are included in the manuscript.

## AUTHORS’ CONTRIBUTIONS

HH, AA, TM, and HI: Conceptualization, study design, supervision, and project administration. ED, HS, and ZNAR: Data collection and investigation. HH, AA, HI, and AH: Literature review, formal analysis, data interpretation, visualization, and original draft preparation. HH, AA, HI, ML, MAAA, and WPL: conducted data interpretation, performed the statistical analysis, manuscript review, and editing. All authors have read and approved the final manuscript.
